# Sero-epidemiological investigation and cross-neutralization activity against SARS-CoV-2 variants in cats and dogs, Thailand

**DOI:** 10.3389/fvets.2024.1329656

**Published:** 2024-05-06

**Authors:** Sarin Suwanpakdee, Natthaphat Ketchim, Metawee Thongdee, Somjit Chaiwattanarungruengpaisan, Siriporn Tangsudjai, Witthawat Wiriyarat, Pruksa Julapanthong, Wachira Trakoolchaisri, Supakit Buamas, Walasinee Sakcamduang, Pilailuk Akkapaiboon Okada, Pilaipan Puthavathana, Weena Paungpin

**Affiliations:** ^1^The Monitoring and Surveillance Center for Zoonotic Diseases in Wildlife and Exotic Animals, Faculty of Veterinary Science, Mahidol University, Nakhon Pathom, Thailand; ^2^Department of Clinical Sciences and Public Health, Faculty of Veterinary Science, Mahidol University, Nakhon Pathom, Thailand; ^3^Department of Pre-Clinical and Applied Animal Science, Faculty of Veterinary Science, Mahidol University, Nakhon Pathom, Thailand; ^4^Prasu-Arthorn Veterinary Teaching Hospital, Faculty of Veterinary Science, Mahidol University, Nakhon Pathom, Thailand; ^5^Department of Medical Sciences, National Institute of Health, Nonthaburi, Thailand; ^6^Center for Research and Innovation, Faculty of Medical Technology, Mahidol University, Nakhon Pathom, Thailand

**Keywords:** cat, dog, SARS-CoV-2, neutralizing antibodies, Thailand

## Abstract

Epidemiological data on SARS-CoV-2 infection in companion animals have been thoroughly investigated in many countries. However, information on the neutralizing cross-reactivity against SARS-CoV-2 variants in companion animals is still limited. Here, we explored the neutralizing antibodies against SARS-CoV-2 in cats and dogs between May 2020 and December 2021 during the first wave (a Wuhan-Hu-1-dominant period) and the fourth wave (a Delta-dominant period) of the Thailand COVID-19 outbreak. Archival plasma samples of 1,304 cats and 1,795 dogs (total = 3,099) submitted for diagnosis and health checks were collected at the Prasu-Arthorn Veterinary Teaching Hospital, Faculty of Veterinary Science, Mahidol University, Nakhon Pathom. A microneutralization test was used to detect neutralizing antibodies against the ancestral Wuhan-Hu-1 and the Delta variants. A plasma sample with neutralizing titers ≥10 was considered positive. Our results showed relatively low seroprevalence with seropositive samples detected in 8 out of 3,099 individuals (0.26, 95% CI 0.11–0.51%). Among these cases, SARS-CoV-2 neutralizing antibodies from both the ancestral Wuhan-Hu-1 and the Delta variants were found in three out of eight cases in two cats (*n* = 2) and one dog (*n* = 1). Furthermore, neutralizing antibodies specific to only the ancestral Wuhan-Hu-1 variant were exclusively found in one cat (*n* = 1), while antibodies against only the Delta variant were detected in four dogs (*n* = 4). Additionally, the neutralizing cross-activities against SARS-CoV-2 variants (Alpha, Beta, and Omicron BA.2) were observed in the seropositive cats with limited capacity to neutralize the Omicron BA.2 variant. In summary, the seropositivity among cats and dogs in households with an unknown COVID-19 status was relatively low in Thailand. Moreover, the neutralizing antibodies against SARS-CoV-2 found in the seropositive cats and dogs had limited or no ability to neutralize the Omicron BA.2 variant. Thus, monitoring SARS-CoV-2 infection and sero-surveillance, particularly in cats, is imperative for tracking virus susceptibility to the emergence of new SARS-CoV-2 variants.

## 1 Introduction

Severe acute respiratory syndrome coronavirus 2 (SARS-CoV-2) was first detected in the Wuhan province of China in late 2019. It has rapidly spread worldwide (1) and the viral genome has acquired new mutations, resulting in the emergence of SARS-CoV-2 variants of concern (VOC) (2). SARS-CoV-2 originated from wild animals, in particular, horseshoe bats (*Rhinolophus affinis*) and Malayan pangolins (*Manis javanica*) (3, 4). SARS-CoV-2 transmission is mainly human-to-human. However, several animal species have been infected with SARS-CoV-2 (5, 6).

Companion animals in close contact with humans (mainly cats and dogs) were thought to be at increased risk of infection (7). SARS-CoV-2 infection in cats and dogs has been investigated to better understand their role in the epidemiology of the disease. SARS-CoV-2 viral isolates (8, 9), SARS-CoV-2 RNA (8–13), and specific SARS-CoV-2 antibodies (8–28) have been detected in cats and dogs in different countries. Previous studies showed that the SARS-CoV-2 virus detected in companion animals corresponds to concurrent locally dominant lineages circulating in the human population (8, 26). The antibody response to the SARS-CoV-2 virus in cats in the United Kingdom followed circulating SARS-CoV-2 variants in humans (28). It indicated that long-term multiple contact between humans and companion animals could cause pathogen spillover. However, it is not clear whether they could become reservoirs for the SARS-CoV-2 virus.

Several studies investigated the role of neutralizing antibodies in viral clearance and protection against SARS-CoV-2 infection/re-infection in cats and dogs, both experimentally and naturally. The seropositive cats demonstrated a robust neutralizing antibody response, effectively preventing re-infection by SARS-CoV-2 (29). In dogs, evidence indicated seroconversion along with neutralizing antibody activities (29). The vaccinated cats had an antibody response that could neutralize the ancestral Wuhan-Hu-1 and the Delta variants at comparable levels (30). Cats and dogs naturally infected with SARS-CoV-2 have produced neutralizing antibodies against the ancestral Wuhan-Hu-1, Alpha, Beta, Delta, and Omicron BA.1 variants, with lower neutralizing antibody titers against the latest variant (26). A similar study was also conducted in Mexico, and the neutralizing antibodies against the ancestral Wuhan-Hu-1 strain found in cats and dogs had a lower capacity to neutralize Omicron BA.1 (31). In humans, the neutralizing antibodies generated in response to SARS-CoV-2 vaccines, based on the ancestral Wuhan-Hu-1 strain, are less effective against the Delta and Omicron variants (32–35). According to a recent animal experiment conducted on Syrian hamsters, it was found that the Omicron BA.2 variant showed a significant level of resistance (2.9-fold) against the convalescent hamster sera previously infected with the Omicron BA.1 variant (36). This highlights the need to investigate the effectiveness of SARS-CoV-2 neutralizing antibodies in naturally infected cats and dogs against other variants of SARS-CoV-2, such as the Omicron BA.2.

To provide the sero-epidemiological data, we investigated SARS-CoV-2 ancestral Wuhan-Hu-1 and Delta variants neutralizing antibodies in 3099 plasma samples from domestic cats and dogs brought for any treatment or health check at the Prasu-Arthorn Veterinary Teaching Hospital, Faculty of Veterinary Science, Mahidol University, Nakhon Pathom during the period from the first to fourth COVID-19 outbreak waves in Thailand, spanning from May 2020 to December 2021. Additionally, samples that tested positive for either or both the ancestral Wuhan-Hu-1 and the Delta variants, were evaluated for cross-reactive neutralizing activity against SARS-CoV-2 variants, including the Alpha, Beta, and Omicron BA.2.

## 2 Materials and methods

### 2.1 Ethical approval

The Faculty of Veterinary Science, Mahidol University-Institute Animal Care and Use Committee (FVS-MU-IACUC) approved the use of animal samples with Animal Ethics No. MUVS-2022-01-01. The study protocol involving the SARS-CoV-2 virus was approved by the Institutional Biosafety Committee of Mahidol University (IBC#2022-050).

### 2.2 Sample collection

The samples used in the study consisted of plasma from cats (*n* = 1,304) and dogs (*n* = 1,795) taken during routine healthcare visits to the Prasu-Arthorn Veterinary Teaching Hospital, Faculty of Veterinary Science, Mahidol University between May 2020 and December 2021. The animals were from 12 provinces in the Central and Western regions of Thailand. Information on species, identity, collection date, sex, age, breed, and location of each animal was recorded (Supplementary Table S1). However, information on the COVID-19 status of their originating households was unavailable. All EDTA blood samples used were residual plasma after routine diagnostic testing. Approximately 1 mL of blood was collected from each animal, and the plasma was separated by centrifugation and stored at −20°C until use. Dog and cat sera (*n* = 40) from 2014 to 2019 (pre-COVID-19 cohort) were from the Monitoring and Surveillance Center for Zoonotic Diseases in Wildlife and Exotic Animals (MoZWE) serum bank, Faculty of Veterinary Science, Mahidol University.

### 2.3 SARS-CoV-2 viruses

The ancestral Wuhan-Hu-1 and Delta SARS-CoV-2 variants were used to detect the neutralizing antibodies in all tested samples. These two strains were selected to match the SARS-CoV-2 strains circulating in humans when the animal blood was collected. Two isolates were included: hCoV-19/Thailand/MUMT-4/2020, representing the ancestral Wuhan-Hu-1 strain (GISAID accession number EPI_ISL_493139), and hCoV-19/Thailand/Nan_SEQ7413/2021, representing the Delta variant (GISAID accession number EPI_ISL_3797061). Three additional SARS-CoV-2 variants were used to detect cross-neutralizing antibodies in the seropositive samples. These were the Alpha, Beta, and Omicron BA.2 variants (hCoV-19/Thailand/Ranong_SEQ4773/2021, GISAID accession number EPI_ISL_3797062.2; hCoV-19/Thailand/Songkhla_SEQ8178/2021, GISAID accession number EPI_ISL_3407848; and hCoV-19/Thailand/NIC_BKK_SEQ4804/2022, GISAID accession number EPI_ISL_9611330). The virus stock was titrated in serial half-log10 dilutions (from 0.5 log to 7 log) to obtain 50% tissue culture infectious dose (TCID50) on 96-well microtiter plates of Vero cells. The infection experiments were performed in a biosafety level-3 (BLS-3) laboratory.

### 2.4 Microneutralization test

All plasma samples were assayed for SARS-CoV-2 ancestral Wuhan-Hu-1 and Delta variant neutralizing antibodies. The in-house microneutralization test (MNT) followed a previously described method (37, 38). An equal volume (60 μL) of serial 2-fold dilutions of heat-inactivated plasma (56°C, 30 min) and 100 TCID50 of SARS-CoV-2 virus were mixed and incubated at 37°C with 5% CO_2_ for 1 h and transferred onto Vero cell monolayers (2 × 10^4^ cells/well). This was maintained for 3 days in Eagle’s minimum essential medium supplemented with 2% fetal bovine serum at 37°C with 5% CO_2_. Each sample was observed for evidence of a cytopathic effect (CPE). To ensure optimal testing results, the viral antigen used in each run was back-titrated, and a positive and negative serum control obtained from a COVID-19 vaccinated and non-vaccinated individual was included in each plate. Each plasma sample was tested in duplicate in 96-well plates. The sample titers were recorded as the reciprocal of the highest serum dilution that neutralized 100% of the tested virus, determined by CPE visualization. Samples with neutralizing antibody titers ≥10 were considered positive. The geometric mean titer (GMT) was estimated for each virus strain in cats and dogs, with neutralizing antibody titers of <10 assigned 5 and ≥ 320 assigned 320.

### 2.5 Statistical analysis

Microsoft Office Excel 2019 was used for data management, and the programming language R version 4.2.2 (R Foundation for Statistical Computing, Vienna, Austria) was used to analyze the GMT and for data analysis.

## 3 Results

**Figure 1:**
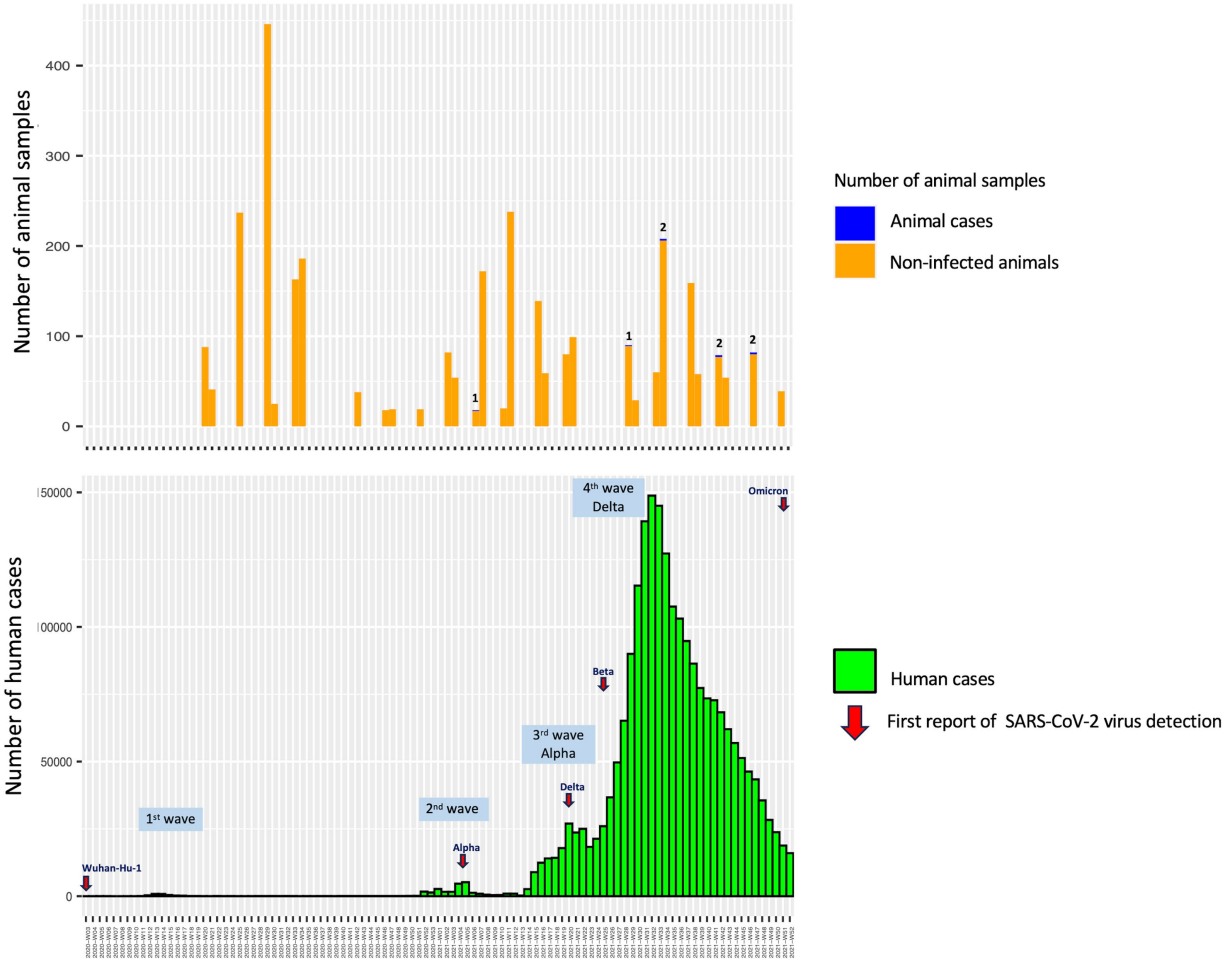
Animal sampling duration and human COVID-19 case detection in Thailand between May 2020 and December 2021.

**Figure 2:**
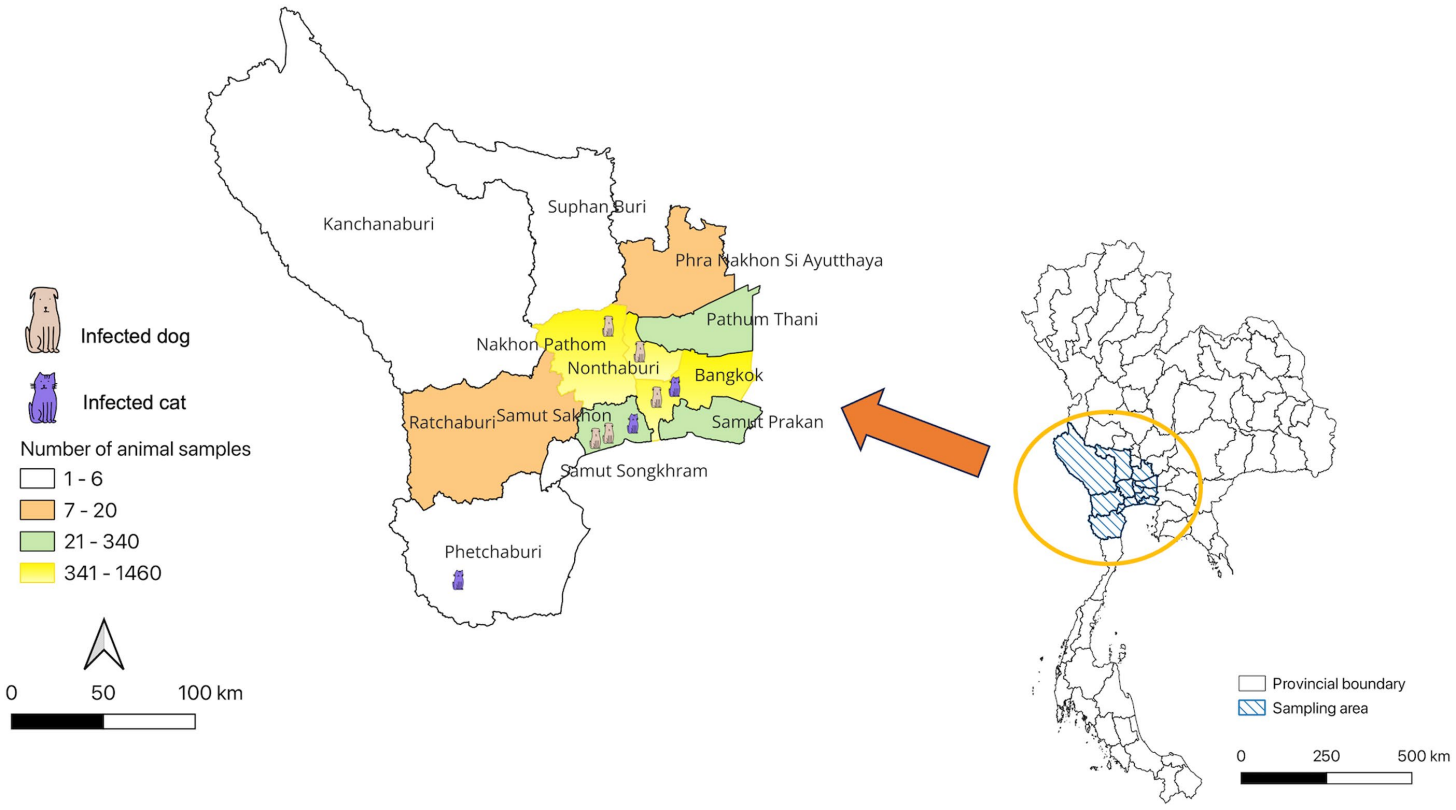
Distribution of SARS-CoV-2 seropositivity cases in cats and dogs, Thailand.

The study revealed that seropositive samples were detected in 8 out of 3,099 individuals (0.26%) (Table 1; Figure 1). The seropositive samples were obtained from three cats (0.23%, 3/1304) and five dogs (0.28%, 5/1795) living in five provinces of Thailand (Table 2; Figure 2). Testing for both pre-COVID-19 sera/plasma from animal samples and those from the first wave of Thai human infections (January–December 2020) and the third wave (April 2021–May 2021) yielded negative results (Figure 1). However, plasma collected from one cat (0.08%) during the second wave (mid-December 2020–March 2021), and five dogs and two cats during the fourth wave (June 2021–December 2021) showed positive results for neutralizing antibodies (Figure 1). Sero-epidemiological studies demonstrated that the highest seropositivity (87.5%, 7/8) was found in the Central region of Thailand, specifically in Samut Sakhon (*n =* 3), Bangkok (*n =* 2), Nakhon Pathom (*n =* 1), and Nonthaburi (*n =* 1) provinces, while only one case (12.5%, 1/8) was found in the Western region, specifically in Phetchaburi province (Table 2; Figure 2).

**Table 1:** Screening for the neutralizing antibody titers against SARS-CoV-2 viruses in cats and dogs by microneutralization assay.

Species	Total	Number of positive samples with the neutralizing antibody titers of ≥ 10, and percentage with 95%CI			
		Wuhan-Hu-1	Delta (B.1.617.2)	Wuhan-Hu-1 and Delta (B.1.617.2)	Total
Cat	1,304	1, 0.07% [95% CI 0.009–0.42]	0, 0.00% [95% CI 0.00–0.28]	2, 0.15% [95% CI 0.01–0.55]	3, 0.23% [95% CI 0.04–0.67]
Dog	1,795	0, 0.00% [95% CI 0.00–0.20]	4, 0.22% [95% CI 0.06–0.56]	1, 0.05% [95% CI 0.001–0.31]	5, 0.27% [95% CI 0.09–0.64]
Total	3,099	1, 0.03% [95%CI 0.0008–0.17]	4, 0.12% [95% CI 0.03–0.33]	3, 0.09% [95% CI 0.01–0.28]	8, 0.26% [95% CI 0.11–0.51]

**Table 2:** Demographic information and neutralizing antibody titers of cats and dogs with SARS-CoV-2 seropositivity.

ID	Species	Date of collection (2021)	Age	Sex	Breed	Location	Neutralizing antibodies titer				
							Wuhan-Hu-1	Alpha (B.1.1.7)	Beta (B.1.351)	Delta (B.1.617.2)	Omicron (BA.2)
1495	Cat	10 February	8 M	Female	Thai	Samut Sakhon	40	40	40	40	<10
2332	Dog	29 July	11 Y 9 M	Male	German Shepherd Mix	Samut Sakhon	<10	<10	<10	40	<10
2597	Dog	26 August	2 Y	Male	Thai	Nakhon Pathom	20	<10	<10	20	<10
2622	Cat	30 August	3 Y 1 M	Male	Thai	Phetchaburi	20	<10	<10	<10	<10
2893	Cat	7 October	5 M	Male	Persian	Bangkok	160	40	40	320	20
2903	Dog	11 October	13 Y 2 M	Male	Golden Retriever	Bangkok	<10	<10	<10	20	<10
2980	Dog	4 November	7 Y 3 M	Female	Thai Ridgeback	Samut Sakhon	<10	<10	<10	20	<10
2981	Dog	4 November	16 Y 4 M	Male	Shih Tzu	Nonthaburi	<10	<10	<10	10	<10
NC	Human	28 December	42 Y 4 M	Female	–	Nakhon Pathom	<10	<10	<10	<10	<10
PC	Human	27 July	32 Y 6 M	Female	–	Samut Prakan	320	20	<10	20	<10

ID, identity; NC, negative control; PC, positive control; M, month; Y, year.

Of eight seropositive samples, three had antibodies against both Wuhan-Hu-1 and Delta variants, including: (1) two seropositive cats (ID 1495 and ID 2893) with neutralizing antibody titers ranging from 40 to 320 (GMT of the ancestral Wuhan-Hu-1 strain = 80 and GMT of the Delta variant = 113) and (2) one seropositive dog (ID 2597) had the neutralizing antibody titers of 20 (GMT of the ancestral Wuhan-Hu-1 strain = 20 and GMT of the Delta variant = 20) (Table 2; Supplementary Table S1). In comparison, we detected neutralizing antibodies specific to only one strain of the SARS-CoV-2 virus in the other five animals. One seropositive cat (ID 2622) (0.08%, 1/1304) had the neutralizing antibodies against only the ancestral Wuhan-Hu-1 strain with titers of 20 (GMT = 20), while four seropositive dogs (ID 2332, ID 2903, ID 2980, and ID 2981) had the neutralizing antibodies against only the Delta variant with titers ranging from 10 to 40 (GMT = 20) (Table 2; Supplementary Table S1).

We further investigated the cross-neutralizing activity of antibodies from seropositive cats and dogs (*n* = 8) against SARS-CoV-2 variants, including the Alpha, Beta, and Omicron BA.2 variants. Our results demonstrated that the seropositive cat (ID 1495) had neutralizing antibody titers of 40 against the ancestral Wuhan-Hu-1, Alpha, Beta, and Delta variants but none against the Omicron BA.2 variant. The seropositive cat (ID 2893) had neutralizing antibody titers of 160, 40, 40, 320, and 20 against the ancestral Wuhan-Hu-1, Alpha, Beta, Delta, and Omicron BA.2 variants, respectively. In contrast, the seropositive dog (ID 2597) had neutralizing antibody titers of 20 against the ancestral Wuhan-Hu-1 and Delta variants but none against the Alpha, Beta, and Omicron BA.2 variants. The other five seropositive animals, including cats (ID 2622) and dogs (ID 2332, ID 2903, ID 2980, and ID 2981), had no neutralizing antibody titers against the tested variants (Table 2).

## 4 Discussion

SARS-CoV-2 virus continues to evolve and adapt to the human population, leading to the emergence of new variants (2). Moreover, the SARS-CoV-2 virus has been transmitted to several animal species, some of which may serve as viral reservoirs (39). Cats and dogs are among the animal species frequently in close contact with humans. Therefore, it is essential to provide the sero-epidemiological data and the cross-reactive neutralizing activity against SARS-CoV-2 variants in these species. This study is a large-scale investigation of SARS-CoV-2 neutralizing antibodies against the ancestral Wuhan-Hu-1 and Delta variants in cats and dogs performed over a prolonged period before the Thailand Omicron variant outbreak.

Our study indicated a relatively low seroprevalence, similar to the other large-scale study in cats and dogs from households with unknown COVID-19 status (14, 17, 23). However, several studies demonstrated that the seropositivity was significantly greater in cats and dogs from COVID-19-positive households compared to those with owners of COVID-19-negative households or unknown status (8, 15, 18, 20, 26).

Spillover of the SARS-CoV-2 virus from humans to pet animals has been continuously documented (8–28). Several studies have demonstrated that SARS-CoV-2 viruses detected in companion animals correspond to concurrent locally dominant lineages circulating in the human population (8, 26). In this study, we found that the prevalence of antibodies in these cats and dogs was also in line with that found in the human population of Thailand, consistent with a previous report (28). Previous sero-epidemiological studies have yielded data indicating a low infection rate of SARS-CoV-2 in Thai people in various risk groups in Bangkok and Chiang Mai Province of Thailand during the first year of the COVID-19 pandemic, which spanned the first two waves and part of the third wave of the COVID-19 outbreaks in Thailand (40). It is consistent with the negative results obtained from animal samples collected during those same periods (Figure 1). Among the seropositive samples, one cat (0.08%) was identified in Samut Sakhon province of Thailand during the second wave, which was considered an epicenter of COVID-19 outbreaks in the country (41). The study by Jairak et al. (42) demonstrated that cats and dogs in Samut Sakhon province of Thailand during the second wave had anti-N-IgG antibodies against SARS-CoV-2, with a prevalence of 3.14% (5/159) as determined by indirect multispecies ELISA. In our study, the highest seroprevalence found in cats and dogs in the fourth wave of COVID-19 outbreaks in Thailand could be linked to the locally dominant viral lineages circulating in the Thai human population, which are primarily driven by the Delta variant (43). It has been reported that the Delta variant is approximately twice as transmissible as the ancestral strain from Wuhan in the human population (44). The study by Jairak et al. (45) demonstrated that cats and dogs in Bangkok and the vicinities of Thailand during the fourth wave were infected with the Delta variant. After viral RNA detection, SARS-CoV-2 antibodies could be detected in both dogs (day 9) and cats (day 14) (45). Although the source of the SARS-CoV-2 infection in cats and dogs in our study is unknown, it is most likely that they contracted the virus from their owners, who were infected during close contact activities.

We further investigated the neutralizing cross-activities against SARS-CoV-2 variants in seropositive cats and dogs. The timing of animal blood sample collection, during which the animal may have been exposed to owners infected with SARS-CoV-2 variants, could impact antibody titers. For instance, the seropositive cat with ID 1495 displayed neutralizing antibody titers of 40 against the ancestral Wuhan-Hu-1, Alpha, Beta, and Delta variants while exhibiting titers of less than 10 against the Omicron BA.2 variant. This cat was discovered to be seropositive in February 2021, during the second wave of SARS-CoV-2 infections in the human population (43). Given that this period coincided with the ancestral Wuhan-Hu-1 variant in the COVID-19 wave, it is possible that the animal was also exposed to this strain. Another seropositive cat with ID 2893 was sampled in October 2021, during the period when the Delta variant was predominant among humans in Thailand (43). The seropositive cat exhibited the highest neutralizing antibody titers of 320 against the dominant Delta variant, compared to other variants (Table 2; Supplementary Table S1). It is important to consider the timing of animal blood sample collection in relation to potential exposure to owners infected with the Delta variant. This seropositive cat showed neutralizing cross-reactivity with similar neutralizing antibody titers (range 40–160) against the ancestral Wuhan-Hu-1, Alpha, and Beta variants and lower neutralizing antibody titers (equal 20) against the Omicron BA.2 variant. Our data are consistent with previous reports showing that cats and dogs were found to produce neutralizing antibodies against the ancestral strain (31) and the Alpha, Beta, Delta, and Omicron BA.1 variants, with lower neutralizing antibody titers against the latter (26, 31).

It is worth noting that the seropositive dog (ID 2597) was sampled in August 2021, during the Delta variant’s dominant period (43). This seropositive dog exhibited neutralizing antibody titers of 20 against both the ancestral Wuhan-Hu-1 and Delta variants, showing no cross-reactivity against the Alpha, Beta, and Omicron BA.2 variants (Table 2). These findings suggest that this dog was previously exposed to the Delta variant.

Several studies demonstrated the difference in SARS-CoV-2 susceptibility experimentally in cats and dogs, which may explain the differences in humoral immune responses against SARS-CoV-2 variants seen in this study. Only SARS-CoV-2-infected cats exhibited viral shedding and tissue tropism, which suggests that cats are highly susceptible to SARS-CoV-2. However, dogs have relatively low susceptibility to SARS-CoV-2 (29, 46). Cats have a higher presence of ACE2 host cell receptors in the respiratory tract than dogs, which may account for more efficient viral replication (47). Younger cats are more susceptible to SARS-CoV-2 than older ones (46). In our study, young cats developed a robust humoral immune response with neutralizing antibody titers ranging from 40 to 320 in two seropositive cats at the age of 5 months (ID 2893) and 8 months (ID 1495). In contrast, the seropositive cat aged 3 years (ID 2622) exhibited low neutralizing antibody titers of 20 (Table 2; Supplementary Table S1). The seropositive cats had neutralizing antibody titers ranging from 20 to 320, while seropositive dogs had antibody titers ranging from 10 to 40. In this study, we found that cats had higher neutralizing antibody titers than dogs, consistent with previous reports (15, 16, 19, 21, 31).

Our study had some limitations: First, all plasma samples were obtained from households with an unknown COVID-19 status. Therefore, we could not determine the impact of disease transmission between humans and animals. Second, more data and positive samples will help determine the accurate level of neutralizing antibodies that cross-react against SARS-CoV-2 variants in cats and dogs. Finally, this was a cross-sectional study relying on single blood samples. Conducting a longitudinal study would be beneficial for gaining a deeper understanding of the persistence of SARS-CoV-2 neutralizing antibodies in cats and dogs under natural conditions. As time passes, neutralizing antibody levels might decrease or disappear in some animals.

In conclusion, the investigation of SARS-CoV-2 neutralizing antibodies has enhanced our understanding of sero-epidemiological data in cats and dogs over an extended period preceding the outbreak of the Omicron variant in Thailand. It was observed that the neutralizing antibodies against SARS-CoV-2 in the seropositive cats and dogs demonstrated limited or no ability to neutralize the Omicron BA.2. Disease surveillance in companion animals, particularly cats, should be maintained due to the possibility of increased susceptibility to new SARS-CoV-2 variants. This could lead to the creation of potential viral reservoirs and transmission between humans and animals.

## Data availability statement

The original contributions presented in the study are included in the article/Supplementary material, further inquiries can be directed to the corresponding author.

## Ethics statement

The animal studies were approved by the Faculty of Veterinary Science, Mahidol University-Institute Animal Care and Use Committee (FVS-MU-IACUC). The studies were conducted in accordance with the local legislation and institutional requirements. Written informed consent was not obtained from the owners for the participation of their animals in this study because All EDTA blood samples were residual samples obtained after routine diagnostic testing. The Institute Animal Care and Use Committee (IACUC) does not require informed consent. However, researchers must submit a protocol for the exemption review when using secondary samples.

## Author contributions

SS: Conceptualization, Writing – original draft, Writing – review & editing, Formal analysis, Visualization, Data curation, Project administration. NK: Writing – review & editing, Investigation, Methodology, Validation, Formal analysis. MT: Methodology, Validation, Investigation, Formal analysis, Writing – review & editing. SC: Methodology, Validation, Investigation, Formal analysis, Writing – review & editing. ST: Project administration, Resources, Investigation, Writing – review & editing. WW: Project administration, Conceptualization, Writing – review & editing. PJ: Data curation, Resources, Investigation, Software, Writing – review & editing. WT: Data curation, Resources, Investigation, Software, Writing – review & editing. SB: Resources, Investigation, Software, Writing – review & editing. WS: Resources, Project administration, Visualization, Writing – review & editing. PO: Project administration, Resources, Writing – review & editing. PP: Project administration, Resources, Writing – review & editing. WP: Project administration, Writing – original draft, Conceptualization, Formal analysis, Methodology, Supervision, Writing – review & editing.
